# National and Regional Medicare Spending on Tafamidis, 2019-2021

**DOI:** 10.1001/jamanetworkopen.2024.26086

**Published:** 2024-09-13

**Authors:** Philip J. Blatt, Timothy Simpson, Hongya Chen, Ahmad Masri

**Affiliations:** 1 Cardiac Amyloidosis Program, Division of Cardiology, Knight Cardiovascular Institute, Oregon Health & Science University, Portland

## Abstract

This cross-sectional study describes national and regional Medicare spending and out-of-pocket costs for tafamidis from its approval in 2019 to 2021.

## Introduction

In May 2019, tafamidis was approved by the US Food and Drug Administration (FDA) for use in transthyretin-amyloid cardiomyopathy (ATTR-CM).^1^ However, at a current list price of $234 900 annually, tafamidis carries a financial burden for the health care system and patients. Additionally, the number of eligible patients for tafamidis is increasing from improved recognition with new imaging modalities.^2^ In 2021, 76.4% of Medicare beneficiaries (approximately 58 million people) had Part D coverage; thus, Medicare data represent the largest subset of the tafamidis prescribing in the US.^3^ In this cross-sectional study, we analyzed Medicare Part D (MPD) claims to understand the total spending and out-of-pocket (OOP) cost of tafamidis from May 2019 through December 2021.

## Methods

For this cross-sectional study, institutional review board oversight and informed consent were waived by the Oregon Health & Science University due to the use public data, which are available through the Centers for Medicare & Medicaid and as such will not be shared by us. This report follows the Strengthening the Reporting of Observational Studies in Epidemiology (STROBE) reporting guidelines.

The data were obtained from the MPD prescribers by geography and drug dataset.^4^ Data from 36 states in 2019, 46 states in 2020, and 47 states in 2021 were available. Medicare did not report data from states with fewer than 11 claims. MPD spending by state is reported directly by Medicare. OOP spending was calculated by dividing the total beneficiary contribution reported by Medicare per state by the number of 30-day fills per state. Medicare spending per beneficiary was calculated by dividing total MPD spending in each state by the number of Medicare beneficiaries in each state as reported by Centers for Medicare & Medicaid Services. Analyses were conducted using Microsoft Excel version 2016 from March 1 to September 1, 2023.

## Results

In 2019, MPD spent $141 807 132.13 on tafamidis. In 2020, spending totaled $442 904 723.63. In 2021, spending increased to $655 914 311.21, a 32% increase from 2020 (Table). In 2021 Medicare reported spending $167 025.69 per year per beneficiary receiving tafamidis, and tafamidis expenditures accounted for 0.28% of MPD expenses nationally. The mean (SD) OOP cost per 30-day fill in 2019 was $738.34 ($198.49) and $518.90 ($253.46) in 2020. In 2021, the mean (SD) OOP costs per fill was $505.59 ($227.99). Annually, patients paid an estimated $6068 OOP for tafamidis in 2021. The states with the highest MPD expenditures in 2021 were New York ($87.65 million), California ($70.48 million), Massachusetts ($57.01 million), Pennsylvania ($56.71 million), and Florida ($47.39 million) (Figure). Adjusting total tafamidis spending for total Medicare beneficiaries per state showed that in 2021, 3 states and Washington, District of Columbia, spent notably more than the national mean (SD) of $9.11 ($8.16) per Medicare beneficiary on tafamidis: Massachusetts ($41.49); Connecticut ($34.77); Washington, District of Columbia ($26.47); and New York ($23.65).

**Table.** Total Spending and Out of Pocket Costs for Tafamidis 2019-2021

Measure	Total spending, $^a^			Mean (SD)					
				% of Total Medicare Part D spending^b^		Spending per Medicare Beneficiary 2021, $	Out-of-pocket per fill, $^c^		
	2019	2020	2021	2020	2021		2019	2020	2021
National total	141 807 132.13	442 904 723.63	655 914 311.21	0.23 (0.20)	0.28 (0.21)	9.11 (8.16)	770.52 (198.49)	518.90 (250.65)	505.69 (227.99)
Per 12 mos	NA	NA	NA	NA	NA	NA	9246.27	6226.79	6068.23

Abbreviation: NA, not applicable.

^a^ Includes total Medicare spending on tafamidis and tafamidis meglumine.

^b^ Includes total Medicare spending on tafamidis by state divided by total Medicare Part D drug expenditures by state.

^c^ Includes total out of pocket cost reported by Medicare divided by number of 30-day prescription fills.

**Figure.**
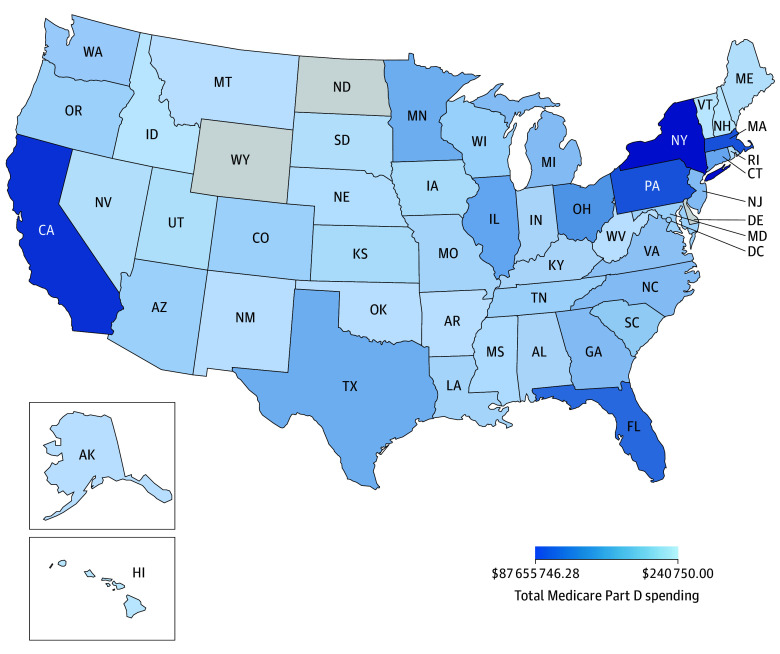
Medicare Part D Spending on Tafamidis by State 2021 Darker color indicates higher spending. No data were available for North Dakota or Wyoming.

## Discussion

This cross-sectional study using the first 2 years of data on Medicare spending on tafamidis found that the total annual cost increased yearly by more than 30% and total spending exceeded $1.2 billion. The expense incurred by Medicare per beneficiary receiving tafamidis at $167 000 annually is substantial, compared with $4362.98 per beneficiary for sacubitril/valsartan.

The mean OOP expense to patients was also notable. Medicare beneficiaries typically have fixed incomes, and the OOP expense is a large portion of their annual income. Total OOP cost annually in 2020 represented 21% of the median income ($29 650) of Medicare beneficiaries. This can lead to overreliance on financial assistance programs, which does not ensure stability of funding.

There are potential cost-saving strategies. For example, the Department of Veterans Affairs dispenses 20 mg daily of tafamidis meglumine instead of the 80 mg of tafamidis meglumine or 61 mg of acid free tafamidis, as approved by the FDA.^5^ While this practice is controversial, it cuts costs by 75%.

The annual spending on tafamidis is expected to increase until a generic form is available or other effective therapies are approved. Within a year of FDA approval of tafamidis, Medicare was spending 0.2% of every MPD dollar on tafamidis. With the increasing recognition of ATTR-CM, demand for tafamidis is expected to increase, and annual cost to MPD could soon exceed $1 billion.

## Notes

Supplement.
Data Sharing Statement
