# Supplementary material for: National and Regional Medicare Spending on Tafamidis, 2019-2021
Source: JAMA Netw Open. 2024 Sep 13;7(9):e2426086. doi: 10.1001/jamanetworkopen.2024.26086 (PMC11400219; doi:10.1001/jamanetworkopen.2024.26086)
Supplement: Supplement. — Data Sharing Statement [file jamanetwopen-e2426086-s001.pdf]

Data Sharing Statement

Blatt. National and Regional Medicare Spending on Tafamidis, 2019-2021. *JAMA Netw Open*. Published September 13, 2024. doi:10.1001/jamanetworkopen.2024.26086

Data
Data available: No

Additional Information
Explanation for why data not available: Data is publicly available online
